# Identification of a vascular invasion-related signature based on lncRNA pairs for predicting prognosis in hepatocellular carcinoma

**DOI:** 10.1186/s12876-023-03118-2

**Published:** 2024-01-15

**Authors:** Nan Zhao, Chunsheng Ni, Danfang Zhang, Na Che, Yanlei Li, Xiao Wang

**Affiliations:** 1 Department of Pathology, Tianjin Medical University, No.22 Qixiangtai Road, Heping District, Tianjin, 300070 China; 2 Department of Pathology, General Hospital of Tianjin Medical University, Tianjin, 300052 China

**Keywords:** Vascular invasion, lncRNA pairs, Prognosis, HCC

## Abstract

**Objectives:**

Most signatures are constructed on the basis of RNA or protein expression levels. The value of vascular invasion-related signatures based on lncRNA pairs, regardless of their specific expression level in hepatocellular carcinoma (HCC), is not yet clear.

**Methods:**

Vascular invasion-related differentially expressed lncRNA (DElncRNA) pairs were identified with a two-lncRNA combination strategy by using a novel modeling algorithm. Based on the optimal cutoff value of the ROC curve, patients with HCC were classified into high- and low-risk subgroups. We used KM survival analysis to evaluate the overall survival rate of patients in the high- and low-risk subgroups. The independent indicators of survival were identified using univariate and multivariate Cox analyses.

**Results:**

Five pairs of vascular invasion-related DElncRNAs were selected to develop a predictive model for HCC. High-risk subgroups were closely associated with aggressive clinicopathological characteristics and genes, chemotherapeutic sensitivity, and highly expressed immune checkpoint inhibitors.

**Conclusions:**

We identified a signature composed of 5 pairs of vascular invasion-related lncRNAs that does not require absolute expression levels of lncRNAs and shows promising clinical predictive value for HCC prognosis. This predictive model provides deep insight into the value of vascular invasion-related lncRNAs in prognosis.

**Supplementary Information:**

The online version contains supplementary material available at 10.1186/s12876-023-03118-2.

## Introduction

Hepatocellular carcinoma (HCC) is the fourth most fatal malignancy. HCC is a complex and multistep disease involving genetic and epigenetic alterations. The etiology and molecular mechanism of HCC remain largely unknown. Although progress has been made in its treatment, the prognosis of HCC is still unsatisfactory because of its extreme heterogeneity. Vascular invasion is associated with worse outcomes in hepatocellular carcinoma (HCC) [1]. Both microscopic and macroscopic vascular invasion are associated with tumor recurrence and short survival times [2]. The increased rate of HCC recurrence is partially caused by microvascular invasion (MVI) [3].

Growing evidence has suggested that long noncoding RNAs (lncRNAs) play a critical role in the development and progression of HCC. It has been demonstrated that numerous lncRNAs associated with HCC are abnormally expressed and contribute to malignant characteristics [4]. LncRNAs, whose transcripts contain more than 200 nt, can regulate gene expression. According to the progress in transcriptome sequencing over the past ten years, we know that more than 70% of the genome is transcribed, and the vast majority of the genome encodes lncRNAs [5]. LncRNAs play a significant role in numerous biological regulatory systems. As a result, LncRNAs are significantly linked to the tumorigenesis, progression, and spread of malignancies [6]. In addition, numerous studies have identified that lncRNAs can alter the intrinsic properties of tumor cells to remodel the tumor microenvironment [7].

Increasing evidence has revealed that signatures related to vascular invasion show promising predictive value for the diagnosis, prognosis and treatment response evaluation of malignant tumors. Moreover, lncRNAs greatly contribute to the development of these signatures. Regrettably, the majority of signatures seem to be constructed based on the absolute expression values for individual RNAs or proteins. However, the accuracy and sensitivity of cancer diagnosis models can be improved by utilizing gene pairs [8].

In the current work, we adopted a two-lncRNA combination strategy that does not require the absolute expression levels of lncRNAs to construct a lncRNA pair signature that correlates with vascular invasion. A signature based on 5 pairs of vascular invasion-related lncRNAs was constructed by using a novel modeling algorithm. Moreover, the risk score generated based on the signature was assessed for its correlation with diverse features, such as survival status, clinicopathological characteristics and chemotherapeutic efficacy.

## Materials and methods

### Data collection (TCGA-LIHC cohort) and differentially expressed analysis

The data including the clinical and RNA sequencing of 365 cases with HCC prior to 13 October, 2021, were obtained from the TCGA website (https://portal.gdc.cancer.gov/repository). The TCGA databases provide publicly accessible data. As a result, the current research was free from requiring a consent of a local ethics commission. The present study complies to TCGA publishing and data access rules. Ensembl (http://asia.ensembl.org) GTF files were obtained for annotation in order to discriminate between mRNAs and lncRNAs for further study. A genes set associated with vascular invasion was obtained from the GSEA dataset (M41805) and utilized to select lncRNAs associated with vascular invasion with a co-expression methodology. We used correlation analysis to explore the lncRNAs related to vascular invasion. LncRNAs were confirmed to be correlated with vascular invasion when the correlation coefficients larger than 0.4 and P values less than 0.001. We utilized the R package limma to do differential expression analysis within vascular invasion-related lncRNAs to determine the differentially expressed lncRNAs (DElncRNA). The cutoffs were defined at false discovery rate (FDR) 0.05 and log fold change (FC) > 2.

### Construction of DElncRNA pairs

We established a 0-or-1 matrix by cyclically individually pairing DElncRNAs as followings: If lncRNA B has a lower level of expression than lncRNA A, then X is regarded as 1, else it is 0. Afterward, the 0-or-1 matrix was subjected to secondary screening. It was regarded a satisfactory match unless the expression quantities of 0 or 1 of lncRNA pairs accounted for greater than 20% of all matches.

### Constructing a predictive model

Vascular invasion-related DElncRNAs having prognostic significance were identified using a univariate Cox analysis of overall survival (OS). This study adopted the least absolute shrinkage and selection operator (LASSO)-penalized Cox regression analysis to confirm a predictive model and reduce the possibility of overfitting. The "glmnet" R package was utilized for variable selection and shrinkage using the LASSO strategy.

The normalized expression levels of all gense and their matching regression coefficients were used to generate the risk scores for the patients. The following formula was developed: score = e^sum (each pairs’ expression×corresponding coefficient)^. Based on the optimal ROC cut-off value, the patients were classified to high-and low-risk subsubgroups.

### Validation of the predictive model

We used the "survminer" R package and survival analysis to compare the overall survival (OS) of patients in high- and low-risk subsubgroups. Time-dependent receiver operating characteristic (ROC) curve studies were performed using the "survival ROC" R package to evaluate the gene signature's predictive ability. We conducted univariate and multivariate Cox regression analyses to identify if it is a favorable modle as an independent factor to predict prognosis. The R packages including survival, pHeatmap, and ggupbr were adopted in the process.

### Evaluation of the significance of the model in the antitumour drugs

IC50 of commonly administered chemotherapeutic medicines in LIHC dataset from TCGA were assessed to evaluate the model's clinical applicability for treating patients with HCC. According to AJCC recommendations, sorafenib and other antitumor medications can be used to treat liver malignacy. We used Wilcoxon signed-rank test to assess the difference of IC50 between the high- and low-risk subsubgroups. The outcomes are presented as box plots through R's pRRophetic and ggplot2 packages.

### Immune components of C1-C6

Immune components of C1-C6 were identified according to “dataset: phenotype—Immune subtype” from UCSC Xena (hub: https://pancanatlas.xenahubs.net).

### Statistical analysis

To evaluate the proportions, chi-squared analysis was employed. KM analysis was used to examine the variations in OS between the subgroups,. The independent variables for OS were screened adopting univariate and multivariate Cox analysis. Spearman or Pearson correlation analysis were performed to determine if the predictive risk score or prognostic gene expression level associated with the drug sensitivity. We made plots adopting R software (Version 4.0.5) with the programs Venn, igraph, ggplot2, pheatmap, ggpubr, corrplot, and survminer. For all findings, a two-tailed P value of less than 0.05 was determined to be statistically significant.

## Results

### Identification of differentially expressed lncRNA (DElncRNA) pairs

**Fig. 1:**
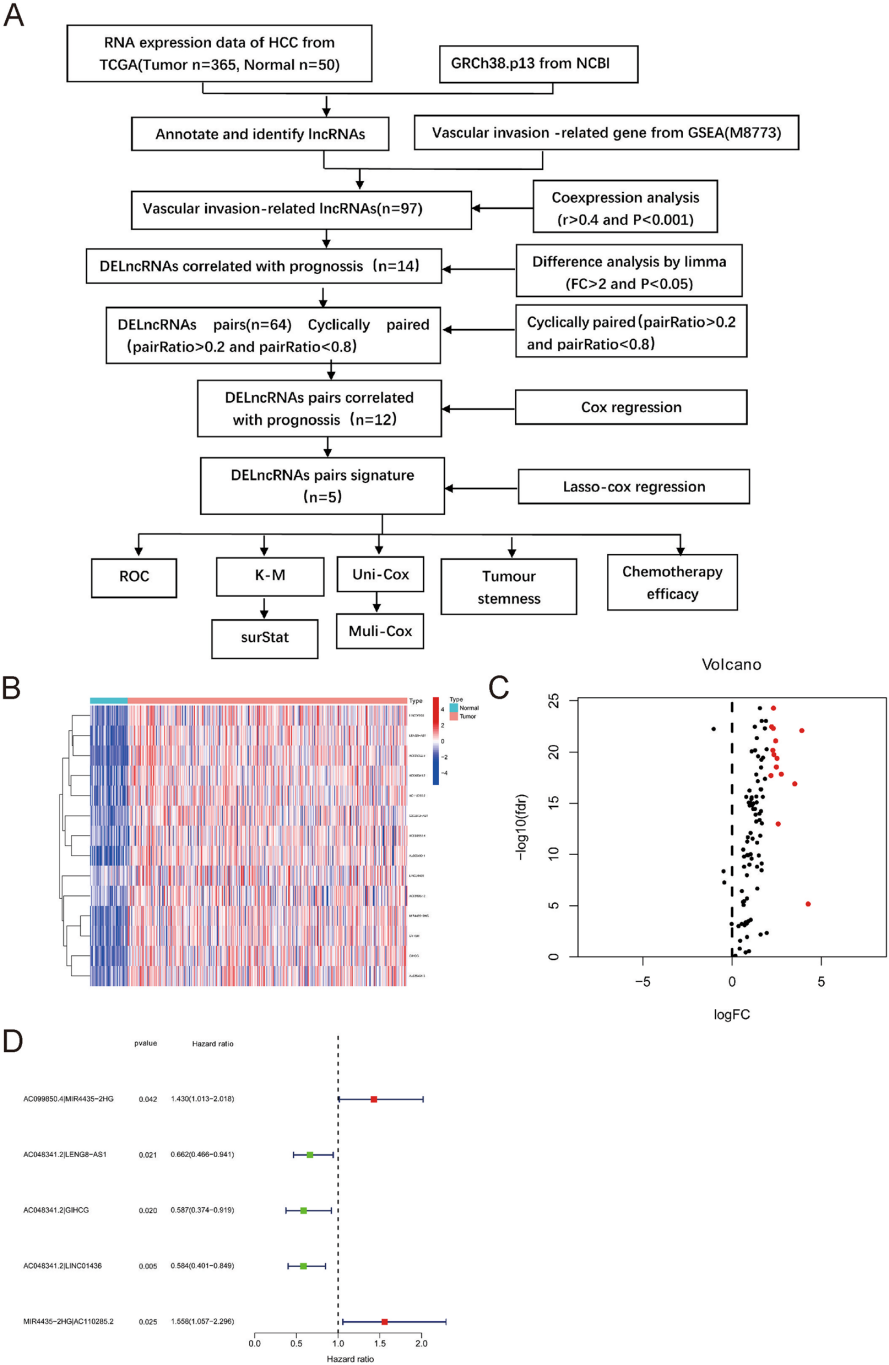
Pairs of Differentially Expressed lncRNAs (DElncRNAs) were identified. **A** Flow chart of the study. The heatmap (**B**) and volcanoplot (**C**) showed that TCGA datasets and Ensembl annotation were used to determine differentially expressed vascular invasion associated lncRNAs. **D** The forest map displayed 5 DElncRNA pairs identified using univariate Cox regression analysis

The process flow is shown in Fig. 1A. The liver hepatocellular carcinoma (LIHC) program of The Cancer Genome Atlas (TCGA) database provided RNA sequencing data of 50 normal and 365 tumour specimens and corresponding patient clinical data (Suppl Table 1). Gene transfer format (GTF) files from Ensembl were applied to annotate the data. Then, a coexpression analysis between vascular invasion-related genes (M8773 from GSEA) and lncRNAs was performed (Suppl Table 2). We identified a total of 97 vascular invasion-related lncRNAs (Suppl Table 3), and 14 DElncRNAs were identified as associated with prognosis (Fig. 1B, Suppl Table 4). All 14 DElncRNAs were upregulated (Fig. 1C).

Next, we constructed 64 valid DElncRNA pairs through an iterative loop and 0-or-1 matrix filtering. Twelve DElncRNA pairs were identified to be vascular invasion-related lncRNA pairs with prognostic significance by univariate Cox analysis (Suppl Table 5). A predictive model was established utilizing LASSO regression analysis, and a signature composed of 5-DElncRNA pairs was identified. This signature was preserved as a prognostic indicator (Fig. 1D).

### Construction of a predictive model for HCC

Based on the optimal value of λ, a signature of 5 DElncRNA pairs was identified (Suppl Fig S1). The risk score was determined based on the following formula: (0.3776)*expression level of AC099850.4|MIR4435-2HG + (-0.3176)*expression level of AC048341.2|LENG8-AS1 + (-0.3402)*expression level of AC048341.2|GIHCG + (-0.3572)*expression level of AC048341.2|LINC01436 + (0.4547)*expression level of MIR4435-2HG|AC110285.2 (Suppl Table 6).

**Fig. 2:**
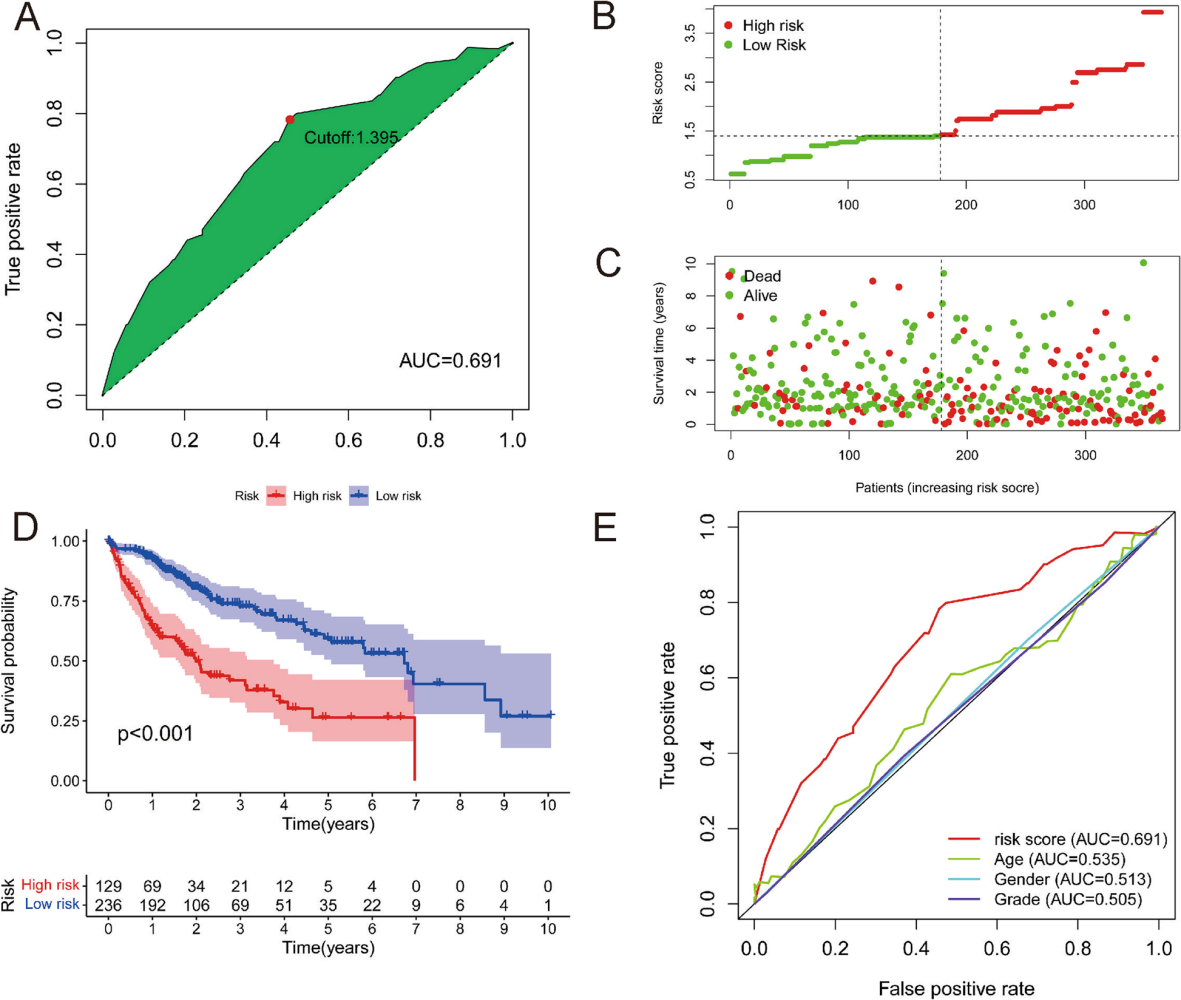
Establishment a and validation of a risk model using DEirlncRNA Pairs. **A** For each receiver operating characteristic (ROC) curve, the areas under curve (AUCs) were evaluated and the curved line were drawn; the cut-off point is the highest inflection point. **B** The distribution and median value of the risk scores in the patients from TCGA; (**C**) The distribution of OS status in the TCGA cohort; (**D**) Kaplan–Meier curves for the individuals from TCGA in the high- and low-risk subsubgroup (**E**) The superiority of the risk score was demonstrated by a comparison of ROC curves with other clinical features

To validate the best DElncRNA pair for obtaining the maximum AUC value, the area under the curve (AUC) values for each receiver operating characteristic (ROC) curve were assessed, and the curve was drawn, with the maximum value pointing to 1.395 (Fig. 2A). Depending on the optimal cut-off value, patients were separated into two subgroups: high-risk (*n* = 187) and low-risk (*n* = 178) (Fig. 2B). The scatter graph shows that patients in the low-risk subgroup had a lower risk of death at early time points than those in the high-risk subgroup (Fig. 2C). Individuals in the low-risk subgroup had considerably better overall survival (OS) than those in the high-risk subgroup according to the K–M survival analysis (Fig. 2D, *P* < 0.001). To validate that the model performed better than other indicators, we compared the ROC curves of the risk score and the clinical characteristics. The results revealed that the risk score had the greatest AUC, suggesting superior prognostic value (Fig. 2E).

### Independent predictive value of the predictive model based on 5 DElncRNA pairs

**Fig. 3:**
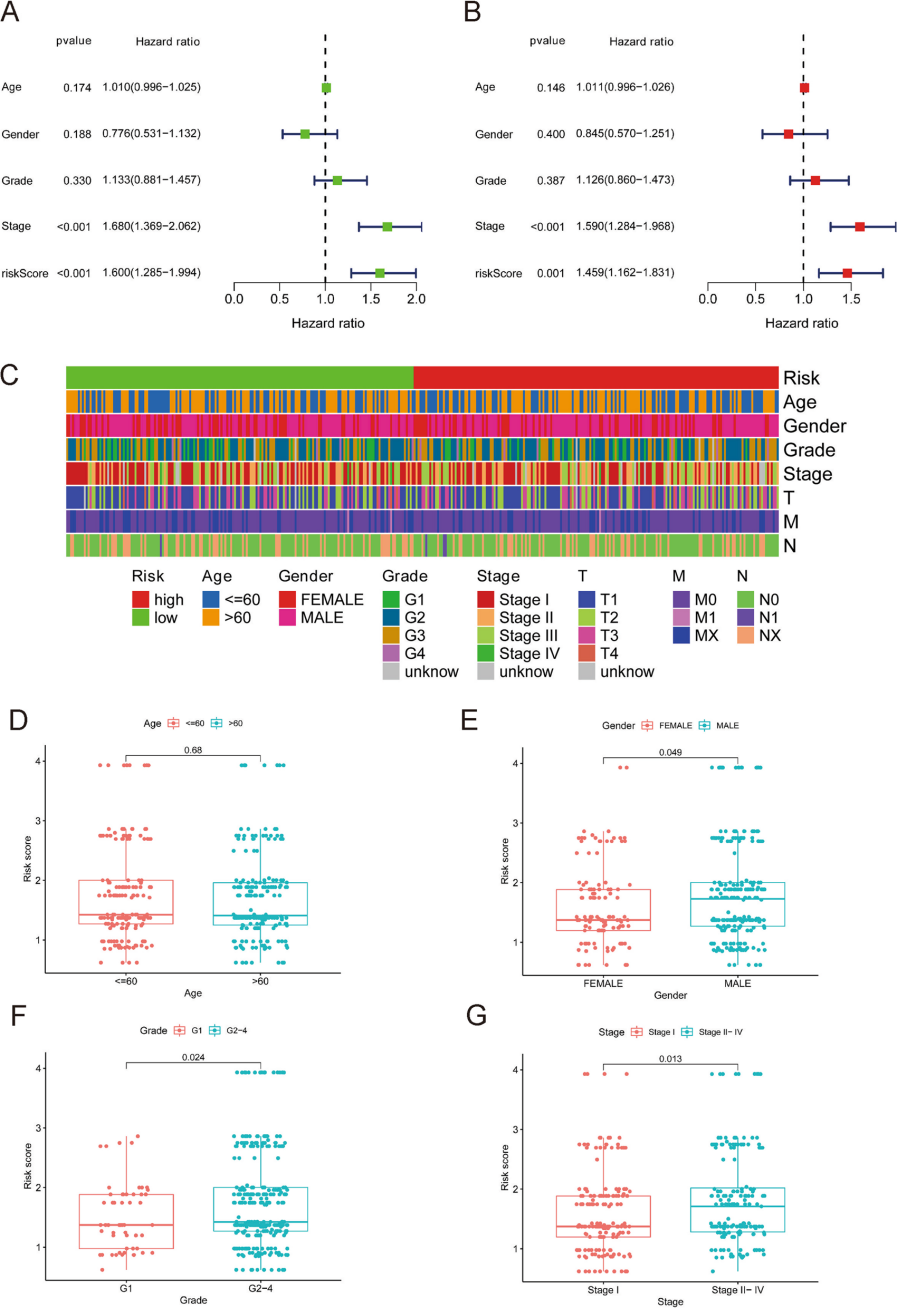
Independent prognostic value of the 5 DElncRNA pairs predictive model. Univariate Cox analyses (**A**) and multivariate Cox analyses (**B**) were used to evaluate survival related characteristics. A strip chart (**C**) and the scatter diagram displaysed (**D**) patients’ age, (**E**) patients’ gender, (**F**) grade and (**G**) stage

Univariate and multivariate Cox analyses were conducted to confirm whether the risk score could act as an independent predictive factor. Univariate Cox analysis of the TCGA cohorts revealed a substantial correlation between OS and the risk score (HR = 1.600, 95% CI = 1.285–1.994, *P* < 0.001) (Fig. 3A). According to multivariate Cox analysis, the risk score was still an independent prognostic factor for patients even when considering other covariates (HR = 1.459, 95% CI = 1.162–1.831, *P* = 0.001) (Fig. 3B).

We then evaluated the relationship between the risk score and the clinical features of HCC patients (Fig. 3C). The outcomes demonstrated that patient sex, tumour grade and tumour stage were apparently associated with the risk score. There was no significant correlation with age (Fig. 3D). As shown in Fig. 3E, males had a much higher risk score than females (*P* < 0.05). The risk score was substantially lower in the grade 1 group than in the grades 2–4 group, as shown in Fig. 3F. (*P* < 0.05). The risk score was substantially lower in stage I than in stage II-IV, as shown in Fig. 3G (*P* < 0.05).

### Association between the levels of immune checkpoint inhibitors,immune components and the risk score of the predictive model

**Fig. 4:**
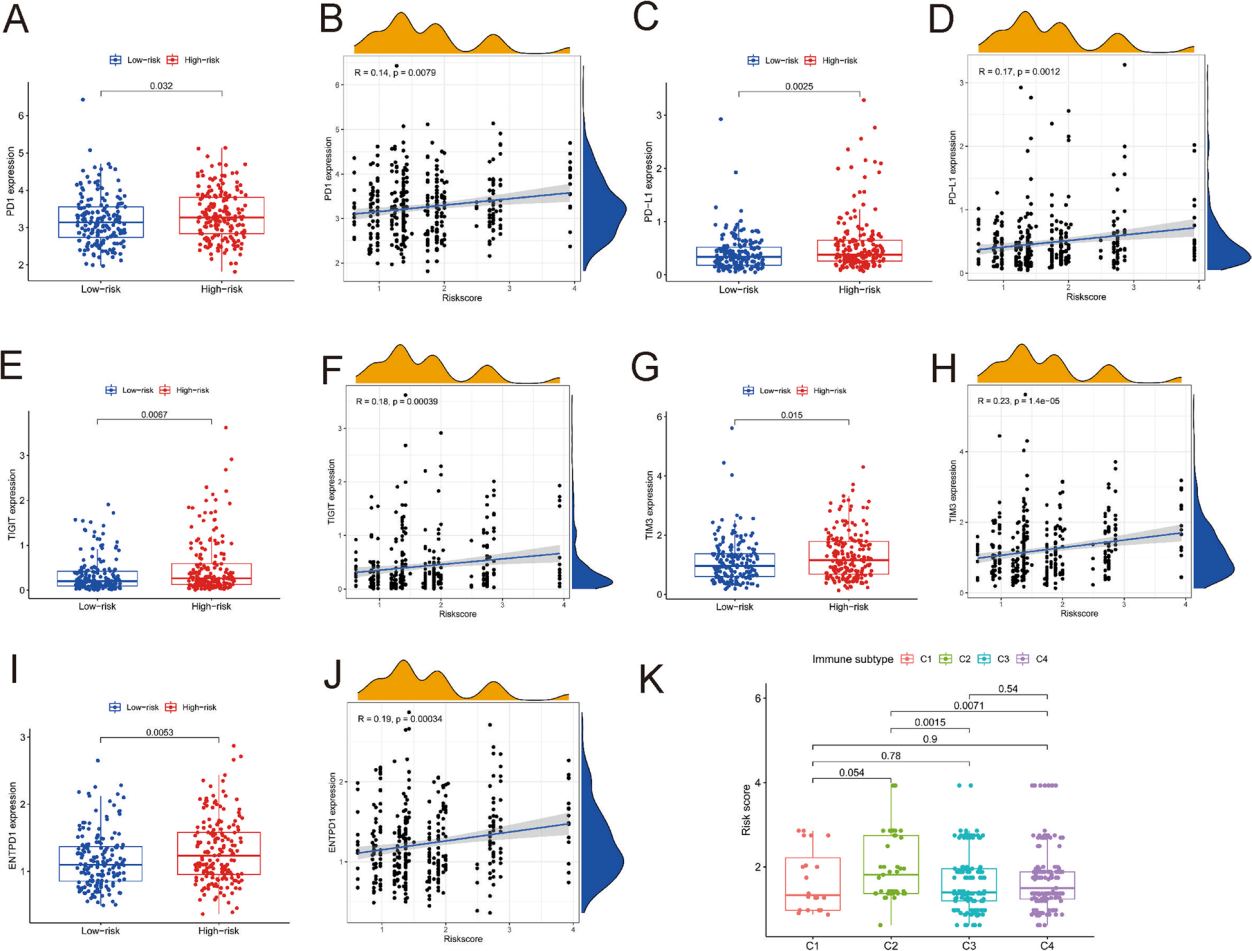
Relationship of immune checkpoint inhibitors, immunological components, and the prediction model. PD1 (**A**), PD-L1 (**C**), TIGIT (**E**), TIM3 (**G**), and ENTPD1 (**I**) expression levels was associated with risk score. The relationship of the level of PD1 (**B**), PD-L1 (**D**), TIGIT (**F**), TIM3 (**H**) and ENTPD1 (**J**) expression and the risk score. Comparison of the risk score in various immune infiltration categories (**K**)

HCC often arises from chronic hepatitis B virus (HBV) infection and does not respond well to immune checkpoint blockade. We investigated the correlations of immune checkpoint inhibitors and risk scores. The results revealed that a low risk score was correlated with decreased expression of PD1 (*P* < 0.05, Fig. 4 A and B), PDL1 (*P* < 0.01, Fig. 4 C and D), TIGIT (*P* < 0.01, Fig. 4 E and F), TIM3 (*P* < 0.05, Fig. 4 G and H) and ENTPD1 (*P* < 0.01, Fig. 4 I and J).

We analysed the effect of the risk score on immune components to detect the relationships between the risk score and immune infiltration type. In human tumours, six kinds of immune infiltrates with phenotypes ranging from tumour-promoting to tumour-suppressive have been recognized [9]; these cell types included C1 (wound healing), C2 (INF-γ dominant), C3 (inflammatory), C4 (lymphocyte depleted), C5 (immunologically quiet) and C6 (TGF-β dominant). None of the specimens in the research contained cells corresponding to the C5 immune subtype or C6 immunological subtype, so the C5 and C6 immune subtypes were excluded from analysis. We investigated the relationship between immune infiltration and the risk score. A low risk score was shown to be closely correlated with C3 and C4 cell subtype, whereas a high risk score was found to be strongly related to the C2 cell subtype (Fig. 4K).

### Relationship of the predictive model and genes

**Fig. 5:**
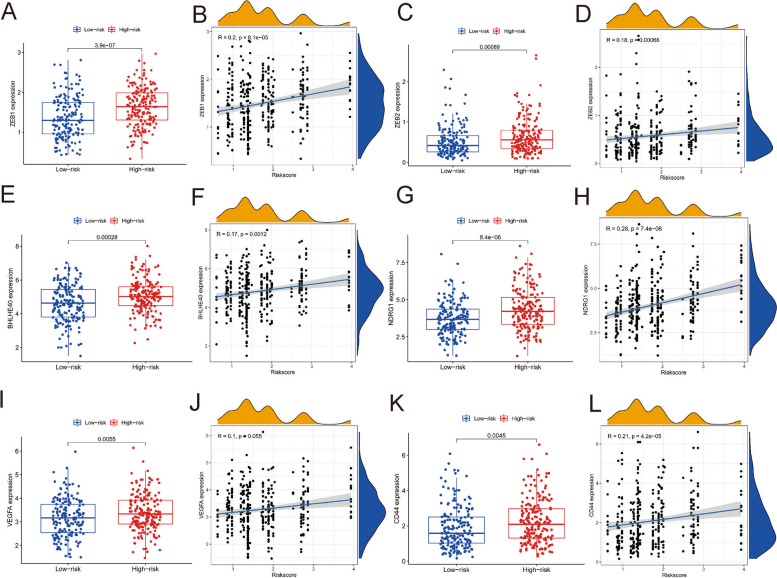
Relationship of the predictive model and genes. The level of of ZEB1 (**A**), ZEB2 (**C**), BHLHE40 (**E**), NDRG1 (**G**), VEGFA (**I**) and CD44 (**K**) expression in both risk subgroups. The relationship of the level of ZEB1 (**B**), ZEB2 (**D**), BHLHE40 (**F**), NDRG1 (**H**), VEGFA (**J**) and CD44 (**L**) expression and the risk score

ZEB1 and ZEB2 are key regulators of epithelial-mesenchymal transition (EMT). The relationship between the risk score and ZEB1 and ZEB2 was analysed. The levels of ZEB1 and ZEB2 expression were obviously higher in the high-risk subgroup than in the low-risk subgroup (Fig. 5A and C). A significant association between the risk score and levels of ZEB1 and ZEB2 expression was recognized (Fig. 5 B and D).

The relationships of risk score with BHLHE40, NDRG1 and VEGFA expression were also studied. The levels of BHLHE40, NDRG1, and VEGFA expression in the low-risk subgroup were substantially lower than those in the high-risk subgroup (Fig. 5E, G and I). BHLHE40, NDRG1, and VEGFA expression levels were all significantly associated with the risk score (Fig. 5 F, H and J).

In numerous solid tumours, CD44 is an important marker for self-renewing cancer stem cells. The relationship between CD44 and the risk score was explored. The level of CD44 expression in the high-risk subgroup was substantially greater than that in the low-risk subgroup (Fig. 5K). CD44 expression levels were apparently related to the risk score (Fig. 5L).

### Relationship of the predictive model and pathway

**Fig. 6:**
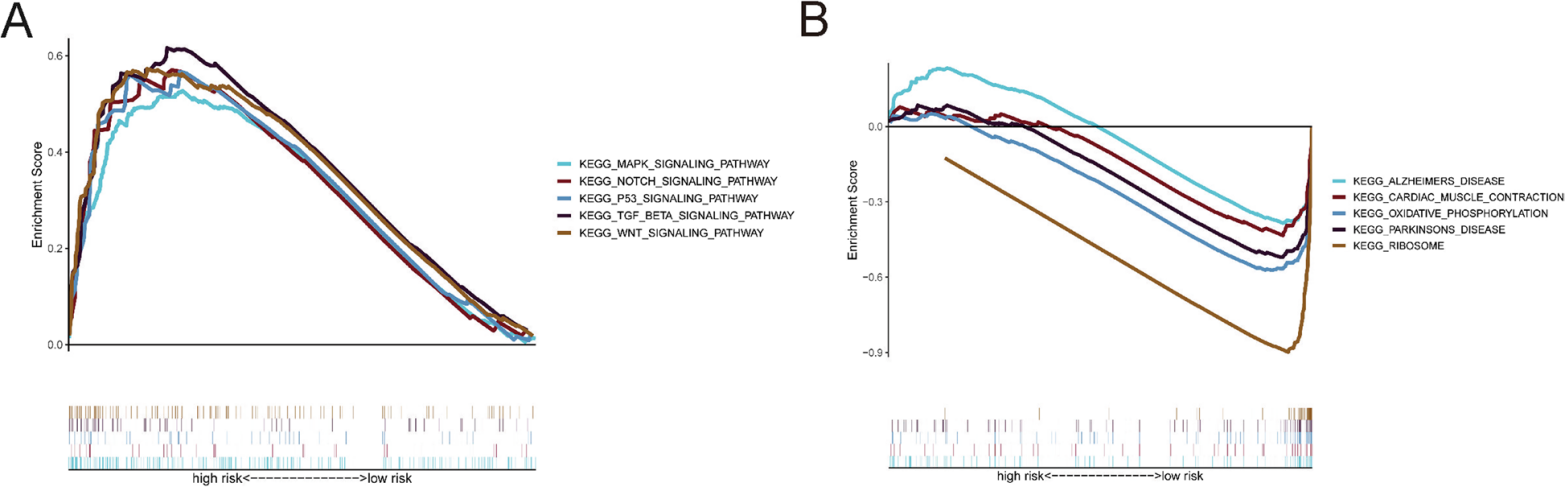
Relationship of the predictive model and pathway. KEGG pathway enrichment investigations between the high-(**A**) and low-risk subsubgroups were carried out using the GSEA (**B**)

We carried out KEGG pathway [10–12] enrichment analysis comparing the high- and low-risk subgroups using GSEA. The high-risk subgroup was shown to have considerably enriched MAPK signalling, NOTCH signalling, TGF-BETA signalling, WNT signalling, and P53 signalling pathways (Fig. 6 A), while the ALZHEIMERS_DISEASE,CARDIAC_MUSCLE_CONTRACTION, OXIDATIVE_PHOSPHORYLATION, PARKINSONS_DISEASE and RIBOSOME signalling pathways were substantially enriched in the low-risk subgroup (Fig. 6B).

### Relationship of the predictive model and chemotherapeutics

**Fig. 7:**
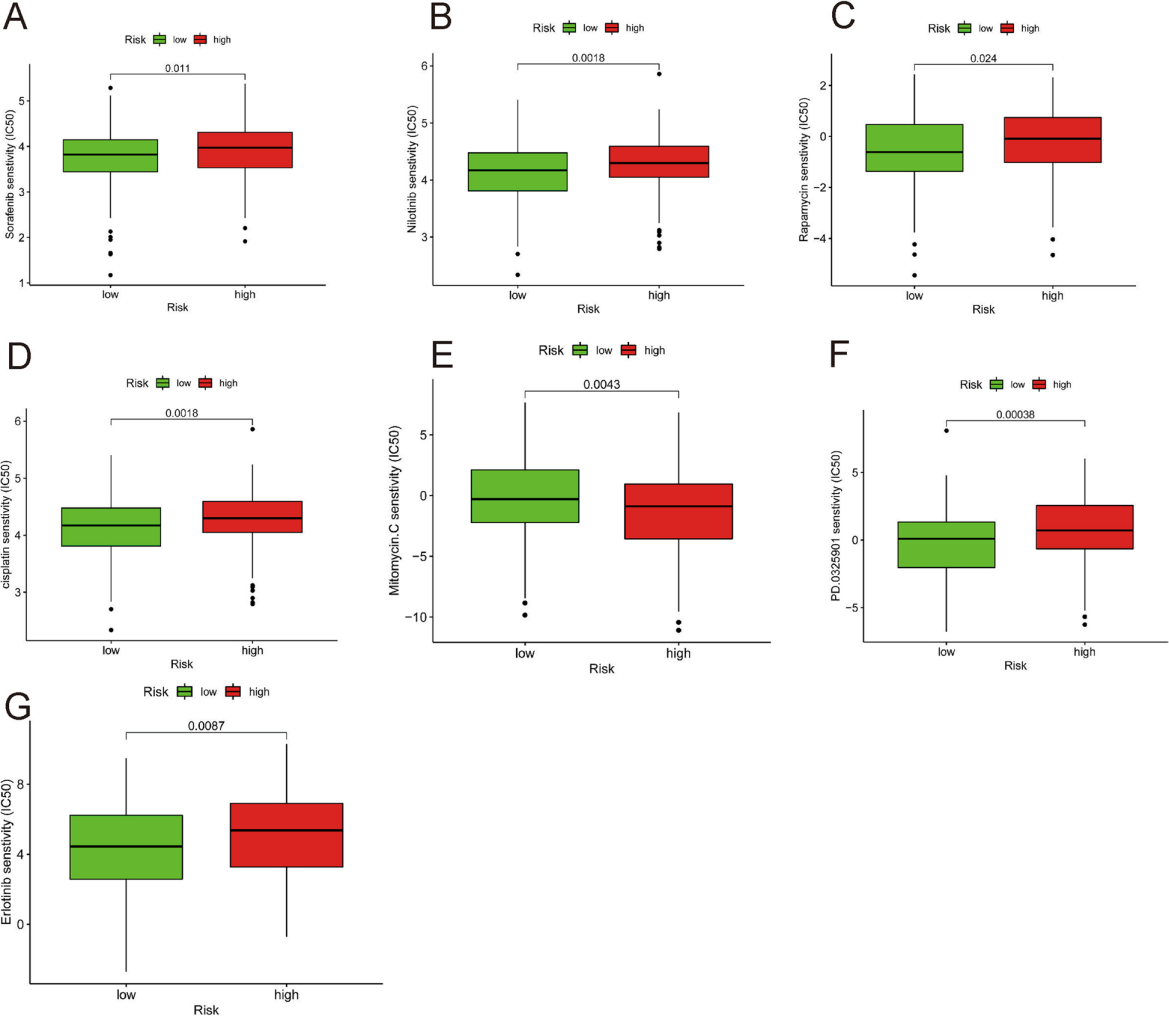
Relationship of predictive model and chemotherapeutics (IC50). (**A**) Sorafenib. **B** Nilotinib. **C** Rapamycin. **D** Cisplatin. **E** Mitomycin.C. **F** PD.0325901. **G** Erlotinib

Common chemotherapeutics are also important for HCC; therefore, the relationship between the risk score and chemotherapy drug sensitivity was also investigated. The results indicated that a high risk score was correlated with a higher half-maximal inhibitory centration (IC50) for chemotherapy drugs such as sorafenib (*P* < 0.05), nilotinib (*P* < 0.01), rapamycin (*P* < 0.05), cisplatin (*P* < 0.01), and mitomycin C (*P* < 0.01), PD.0325901 (*P* < 0.001) and erlotinib (*P* < 0.01), which indicated that the model might be applied to predict chemosensitivity (Fig. 7).

## Discussion

In recent years, an increasing number of studies have aimed to construct signatures to predict the prognosis of patients with malignancies. The absolute expression levels of transcripts need to be detected for most of these signatures. In the present study, a decent perspective model was developed using two-lncRNA combinations, so absolute gene expression values were not needed for the signature. With this two-lncRNA combination model, only the relative expression level of the lncRNA pairs within the data needs to be considered, and there is no need for batch correction of differences between different kinds of data.

Although the relationship between vascular invasion and human cancer has been studied by some researchers, there are few reports on its correlation with immune components. The association between the risk score and immunological components was also investigated to better understand the role of the risk score in immune infiltration. The results showed that a high risk score was highly correlated with enrichment of the C2 cluster, but a low risk score was closely related to enrichment of the C3 and C4 clusters, suggesting that C2 induces tumorigenesis and progression, while C3 and C4 are favorable protective elements. This conclusion was consistent with earlier research since increased cytotoxicity can limit tumor incidence and progression (the immune phenotypes are numbered from 1 to 6 from lowest to highest relative abundance of cytotoxic cells) [9].

Recent studies have improved our understanding of immune checkpoint expression in HCC and have indicated that immune checkpoint blockade could be a rational therapeutic approach even for HCC therapy [13, 14]. High risk scores were shown to be correlated with high levels of PD1, PDL1, TIM3, ENTPD1, and TIGIT. PD-L1 is frequently highly expressed in cancer cells as a defense strategy, as this phenotype facilitates escape from immune surveillance. New treatments targeting immunological checkpoints, such as anti-PD-L1 antibodies, have demonstrated therapeutic effectiveness in a variety of tumors [14]. T-cell exhaustion, characterized by decreased capacity of T cells to release cytokines along with upregulation of immunological checkpoint receptors (for example, PD-1 and CTLA4), has been reported in several tumors, including HCC [15]. The expression levels of the immune checkpoint inhibitory molecules PD-1 and TIM3 in tumor-associated antigen-specific T cells from HCC specimens are higher than those in T cells from tumor-free liver tissues or blood. Strategies to block PD-L1 and TIM3 should be explored for the treatment of HCC.

Epithelial–mesenchymal transition (EMT) is a critical step in tumor progression and metastasis. ZEB1 and ZEB2 are structurally related E-box binding homeobox transcription factors that can promote EMT [16]. To investigate the role of the risk score in EMT, the correlation between ZEB1, ZEB2 and the risk score was examined. The levels of ZEB1 and ZEB2 expression were considerably lower in the low-risk subgroup than in the high-risk subgroup according to the results. The levels of ZEB1 and ZEB2 expression were considerably lower in the low-risk subgroup than in the high-risk subgroup, suggesting that the risk score is a good marker for indicating EMT. In our previous study, we found that VEGFA, NDRG1 and BHLHE40 may suggest the presence of satellite nodules in HCC [17]. To better understand the correlations between the risk score and the satellite nodules, the association between the risk score and VEGFA, NDRG1, and BHLHE40 was also investigated. The findings also suggested that the risk score is an effective indicator. HCC cells possess stem cell-like features, such as immortality, resistance to treatment, and transplantability [18]. CD44 has already been validated as an informative marker of stem cells in primary tumors. To gain more insight into the role of the risk score in tumor stemness, the relationship between the risk score and CD44 was analyzed. The relationships of the risk score and CD44 were investigated to acquire a better understanding of the role of the risk score in tumor stemness. The results showed that CD44 expression was considerably higher in the high-risk subgroup than in the low-risk subgroup. The risk score was positively related to CD44 expression, suggesting that it is a good marker to detect tumor stemness.

Based on pathway analysis, tumor-related signaling pathways, such as the MAPK, NOTCH, TGF-BETA, WNT, and P53 signaling pathways, were considerably enriched in the high-risk subgroup. The involvement of these pathways has been associated with HCC, suggesting novel therapeutic targets [19–21]. The correlation analysis between the predictive model and chemotherapeutics indicated that the risk score was correlated with sensitivity to chemotherapeutics such sorafenib, nilotinib, rapamycin, cisplatin, PD.0325901, and mitomycin C and erlotinib. Sorafenib was the only systemic therapy option for patients with advanced HCC for almost a decade. Nilotinib inhibits MYC and NOTCH1 expression in HCC cell lines, inhibits the growth of xenograft tumors in mice, and inhibits the formation of liver tumors in animals harboring MET and catenin β1 transposons, lowering MYC and NOTCH1 levels in tumors [22]. Rapamycin, an mTOR inhibitor, can reduce the protumorigenic impact of VEPH1 knockdown and is an effective therapeutic option for patients with HCC [23]. Cisplatin is a conventional chemotherapeutic agent. Mitomycin C promotes bystander killing in homogeneous and heterogeneous hepatoma cellular models [24]. Erlotinib inhibits cell cycle progression and causes apoptosis of HCC cells while increasing chemosensitivity to cytostatics [25].

## Conclusion

In summary, this study revealed a novel predictive signature comprised of 5 vascular invasion-related lncRNA pairs. The signature was independently related to OS in patients with HCC and was verified to be effective in functional analysis. The risk score based on this signature was found to be related to the levels of important genes and immune checkpoint inhibitors and chemotherapeutic sensitivity, providing information for predicting HCC prognosis. External validation by other clinical datasets would be helpful, so we will collect new clinical specimens to increase the sample size for further validation in the future. Overall, this study provides promising insight into vascular invasion-related lncRNAs. The signature composed of 5 vascular invasion-related lncRNA pairs does not require the absolute expression values of lncRNAs and could be utilized for HCC diagnosis and prognosis evaluation, which suggests that it is valuable for the development of personalized cancer therapies.

### Supplementary Information

**Additional file 1: Fig S1.** Construction of a prognostic model in HCC cohort. **Suppl Table 1.** Clinical characteristics of the HCC patients used in this study. **Suppl Table 2.** Correlation between vascular invasion-related gene and lncRNA. **Suppl Table 3.** Vascular invasion-related lncRNAs. **Suppl Table 4.** Vascular invasion-related lncRNAs correlated with prognosis. **Suppl Table 5.** Vascular invasion -related lncRNA pairs with prognostic value. **Suppl Table 6.** Vascular invasion -related lncRNA pairs signature.

## Data Availability

All data generated or analyzed during this study are included in this published article. The datasets generated and/or analysed during the current study are available on https://portal.gdc.cancer.gov/.
