# Supplementary material for: Identification of a vascular invasion-related signature based on lncRNA pairs for predicting prognosis in hepatocellular carcinoma
Source: BMC Gastroenterol. 2024 Jan 15;24:33. doi: 10.1186/s12876-023-03118-2 (PMC10788995; doi:10.1186/s12876-023-03118-2)
Supplement: Supplementary file 1 — Additional file 1: Fig S1. Construction of a prognostic model in HCC cohort. Suppl Table 1. Clinical characteristics of the HCC patients used in this study. Suppl Table 2. Correlation between vascular invasion-related gene and lncRNA. Suppl Table 3. Vascular invasion-related lncRNAs. Suppl Table 4. Vascular invasion-related lncRNAs correlated with prognosis. Suppl Table 5. Vascular invasion -related lncRNA pairs with prognostic value. Suppl Table 6. Vascular invasion -related lncRNA pairs signature. [file 12876_2023_3118_MOESM1_ESM.pdf]

Supplement materials

1. Supplement Figure

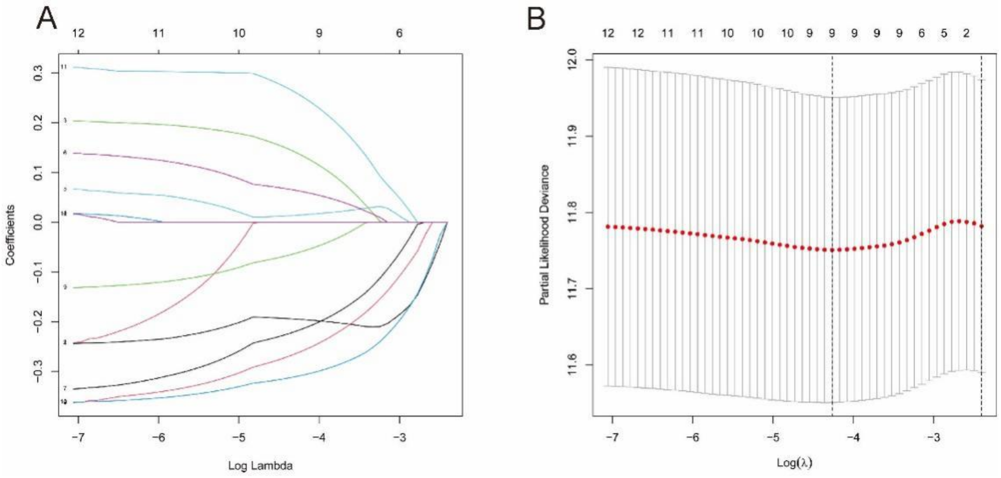

Fig S1 Construction of a prognostic model in HCC cohort. (A) LASSO coefficient expression profiles of candidate genes. (B) The penalty parameter (λ) in the LASSO model was selected through cross-validation.

2. Supplement table

Suppl Table 1 Clinical characteristics of the HCC patients used in this study

Characteristics		No. (Percent)
**No. of patients**		365
**Age(median,range)**		61(16-90)
**Age**	<=60	173(47.4%)
	>60	192(52.6%)
**Gender**	FEMALE	119(32.6%)
	MALE	246(67.4%)
**Grade**	G1	55(15.07%)
	G2	175(47.95%)
	G3	118(32.33%)
	G4	12(3.29%)
	unknow	5(1.37%)
**Stage**	Stage I	170(46.58%)
	Stage II	84(23.01%)
	Stage III	83(22.74%)
	Stage IV	4(1.1%)
	unknow	24(6.58%)
**T**	T_1	180(49.32%)
	T_2	91(24.93%)
	T_3	78(21.37%)
	T_4	13(3.56%)
	T_X	1(0.27%)
	unknow	2(0.55%)
**M**	M_0	263(72.05%)
	M_1	3(0.82%)
	M_X	99(27.12%)
**N**	N_0	248(67.95%)
	N_1	4(1.1%)
	N_X	112(30.68%)
	unknow	1(0.27%)

Suppl Table 2 Correlation between vascular invasion-related gene and lncRNA

Vascular invasion related gene	lncRNA	cor	pvalue	Regulation
SERPINB6	AC073896.4	0.4328	1.68E-18	postive
RPAP1	LINC00205	0.4519	3.23E-20	postive
ZNF687	LINC00205	0.4305	2.63E-18	postive
AMPD3	AC015922.2	0.4338	1.36E-18	postive
CA2	AC093010.2	0.4591	6.83E-21	postive
RPAP1	AC099850.4	0.4834	2.70E-23	postive
ZNF687	AC099850.4	0.4031	4.82E-16	postive
ZNF687	ASB16-AS1	0.5005	4.20E-25	postive
AMPD3	CD27-AS1	0.4557	1.40E-20	postive
SERPINB6	SNHG12	0.4651	1.81E-21	postive
ZNF687	AC005332.6	0.4077	2.09E-16	postive
SERPINB6	HEIH	0.4127	8.14E-17	postive
SERPINB6	AC012510.1	0.4388	4.90E-19	postive
UBE3C	AC012313.1	0.4122	9.06E-17	postive
AMPD3	AC048341.2	0.4433	1.94E-19	postive
SERPINB6	ZFAS1	0.4967	1.07E-24	postive
RPAP1	AC093227.1	0.4099	1.38E-16	postive
ZNF687	AC093227.1	0.4377	6.18E-19	postive
AMPD3	AC138207.5	0.4982	7.47E-25	postive
SERPINB6	MIR4435-2HG	0.4274	4.83E-18	postive
AMPD3	HCG18	0.4215	1.53E-17	postive
RPAP1	HCG18	0.5047	1.44E-25	postive
UBE3C	PAXIP1-AS2	0.6760	2.94E-51	postive
AMPD3	LINC01857	0.4479	7.51E-20	postive
SERPINB6	AC055822.1	0.4107	1.19E-16	postive
TRIM8	USP46-AS1	0.4155	4.84E-17	postive
UBE3C	USP46-AS1	0.4353	9.99E-19	postive
RPAP1	SNHG20	0.4015	6.40E-16	postive
RPAP1	LENG8-AS1	0.4328	1.67E-18	postive
AMPD3	LINC02035	0.5675	2.93E-33	postive
SERPINB6	SNHG17	0.4184	2.78E-17	postive
ZNF687	AL358472.2	0.4045	3.74E-16	postive
AMPD3	AC115618.2	0.4171	3.57E-17	postive
SERPINB6	ZNF529-AS1	0.5001	4.64E-25	postive

SERPINB6	SNHG32	0.4335	1.44E-18	postive
AMPD3	LINC00847	0.4728	3.14E-22	postive
SERPINB6	LINC00847	0.4152	5.15E-17	postive
ZNF687	AC110285.2	0.4355	9.65E-19	postive
CA2	LINC02761	0.4442	1.61E-19	postive
SERPINB6	AC002398.1	0.6024	2.62E-38	postive
OGG1	AC097359.2	0.4552	1.57E-20	postive
AMPD3	HCP5	0.4555	1.47E-20	postive
UBE3C	LINC01278	0.4887	7.50E-24	postive
ZNF687	LINC01278	0.4178	3.10E-17	postive
UBE3C	AC008764.2	0.4599	5.69E-21	postive
SERPINB6	GIHCG	0.4150	5.29E-17	postive
UBE3C	NNT-AS1	0.4206	1.84E-17	postive
AMPD3	SMIM25	0.4420	2.56E-19	postive
OGG1	AC009779.3	0.4067	2.48E-16	postive
UBE3C	AC024075.1	0.5372	2.41E-29	postive
RPAP1	SNHG21	0.4502	4.61E-20	postive
RPAP1	CTBP1-DT	0.5249	7.24E-28	postive
UBE3C	CTBP1-DT	0.4393	4.49E-19	postive
ZNF687	CTBP1-DT	0.5178	4.83E-27	postive
RPAP1	PTOV1-AS1	0.4086	1.76E-16	postive
RPAP1	MID1IP1-AS1	0.4891	6.81E-24	postive
UBE3C	MID1IP1-AS1	0.5585	4.62E-32	postive
AMPD3	NRAV	0.4269	5.38E-18	postive
RPAP1	NRAV	0.4390	4.73E-19	postive
SERPINB6	NRAV	0.4676	1.02E-21	postive
AMPD3	LINC00342	0.4763	1.41E-22	postive
RPAP1	AC067852.2	0.5099	3.78E-26	postive
ZNF687	AC067852.2	0.4568	1.11E-20	postive
AMPD3	TNFRSF10A-AS1	0.4353	1.01E-18	postive
UBE3C	TNFRSF10A-AS1	0.4286	3.82E-18	postive
SERPINB6	AGAP2-AS1	0.4388	4.92E-19	postive
SERPINB6	AC046143.2	0.4428	2.16E-19	postive
AMPD3	AC060766.4	0.4221	1.37E-17	postive
SERPINB6	AC060766.4	0.4165	4.03E-17	postive
ZNF687	C2CD4D-AS1	0.4631	2.80E-21	postive

ZNF687	WAC-AS1	0.4227	1.21E-17	postive
AMPD3	LINC01836	0.4673	1.11E-21	postive
AMPD3	FAM111A-DT	0.5252	6.57E-28	postive
CSF3R	AC068987.3	0.4686	8.17E-22	postive
RPAP1	RAD51-AS1	0.4198	2.11E-17	postive
RPAP1	AC007406.5	0.5253	6.50E-28	postive
ZNF687	AC007406.5	0.4162	4.25E-17	postive
AMPD3	SPINT1-AS1	0.4206	1.84E-17	postive
RPAP1	LINC00909	0.4071	2.33E-16	postive
AMPD3	PCED1B-AS1	0.4906	4.81E-24	postive
AMPD3	AC010547.2	0.4108	1.16E-16	postive
RPAP1	NORAD	0.4751	1.85E-22	postive
UBE3C	NORAD	0.4073	2.24E-16	postive
ZNF687	NORAD	0.4235	1.05E-17	postive
SERPINB6	LINC01436	0.4799	6.13E-23	postive
AMPD3	AP000759.1	0.4232	1.11E-17	postive
ZNF687	AL606489.1	0.5604	2.63E-32	postive
RPAP1	SNHG14	0.4298	3.01E-18	postive
UBE3C	AL450326.1	0.4156	4.71E-17	postive
SERPINB6	AC067838.1	0.4531	2.47E-20	postive
RPAP1	AC125257.1	0.4054	3.17E-16	postive
ZNF687	AC125257.1	0.4601	5.41E-21	postive
CA2	CA3-AS1	0.5447	2.78E-30	postive
ZNF687	AL162595.1	0.4480	7.29E-20	postive
TRIM8	AL391121.1	0.7219	1.89E-61	postive
UBE3C	AL391121.1	0.4855	1.62E-23	postive
SERPINB6	MAPKAPK5-AS1	0.6139	4.21E-40	postive
AMPD3	ANKRD10-IT1	0.4110	1.13E-16	postive
SERPINB6	AL035461.3	0.4716	4.13E-22	postive
CSF3R	CASC19	0.4371	6.94E-19	postive
RPAP1	OIP5-AS1	0.4617	3.81E-21	postive
UBE3C	OIP5-AS1	0.4422	2.43E-19	postive
SERPINB6	CYTOR	0.4315	2.16E-18	postive
UBE3C	EBLN3P	0.5406	9.01E-30	postive
ZNF687	EBLN3P	0.4030	4.87E-16	postive
UBE3C	AC000123.1	0.5207	2.22E-27	postive
ZNF687	AP001318.2	0.4223	1.31E-17	postive

RPAP1	AC009237.14	0.4103	1.29E-16	postive
AMPD3	THAP9-AS1	0.4391	4.61E-19	postive
AMPD3	AP003486.1	0.4006	7.52E-16	postive
AMPD3	AC104083.1	0.4028	5.03E-16	postive
RPAP1	SNHG16	0.4306	2.57E-18	postive
AMPD3	AC005288.1	0.4773	1.13E-22	postive
RPAP1	AC005288.1	0.5936	5.69E-37	postive
UBE3C	AC005288.1	0.6025	2.54E-38	postive
ZNF687	AC005288.1	0.5013	3.39E-25	postive
SERPINB6	AC073611.1	0.4363	8.22E-19	postive
RPAP1	AC074117.1	0.4298	2.99E-18	postive
AMPD3	NRSN2-AS1	0.4947	1.75E-24	postive
RPAP1	FGD5-AS1	0.5210	2.08E-27	postive
UBE3C	FGD5-AS1	0.5278	3.29E-28	postive
ZNF687	FGD5-AS1	0.5031	2.17E-25	postive
AMPD3	EIF3J-DT	0.4401	3.74E-19	postive
SERPINB6	AL035071.1	0.4154	4.93E-17	postive
RPAP1	AC005586.1	0.4958	1.33E-24	postive
UBE3C	AC005586.1	0.5655	5.33E-33	postive
AMPD3	AC011451.1	0.4126	8.34E-17	postive
ZNF687	AC005332.5	0.4107	1.18E-16	postive
AMPD3	AL354836.1	0.4098	1.39E-16	postive

Suppl Table 3 Vascular invasion-related lncRNAs

gene	conMean	treatMean	logFC	pValue	fdr
AC073896.4	3.2027	6.2532	0.9653	6.92E-10	1.00E-09
LINC00205	0.2893	1.4056	2.2803	1.03E-21	6.65E-21
AC015922.2	0.7421	0.5470	(0.4402)	4.18E-08	5.56E-08
AC093010.2	12.9674	14.7532	0.1861	7.84E-01	7.92E-01
AC099850.4	0.1832	2.0816	3.5064	3.81E-18	1.27E-17
ASB16-AS1	0.8352	2.6245	1.6519	2.86E-25	9.09E-24
CD27-AS1	1.3416	2.6298	0.9710	1.89E-17	5.74E-17
SNHG12	0.4736	1.8113	1.9353	7.10E-22	5.30E-21
AC005332.6	5.0821	8.0704	0.6672	1.08E-10	1.64E-10
HEIH	5.3418	12.8664	1.2682	2.07E-24	3.35E-23
AC012510.1	0.2878	0.9491	1.7214	8.56E-17	2.37E-16
AC012313.1	0.6099	1.3048	1.0972	9.70E-17	2.61E-16
AC048341.2	0.2150	1.0879	2.3390	3.03E-21	1.73E-20
ZFAS1	6.4592	19.5377	1.5968	1.38E-17	4.31E-17
AC093227.1	0.4819	0.8567	0.8302	1.56E-06	1.99E-06
AC138207.5	1.2375	1.6571	0.4213	1.54E-01	1.60E-01
MIR4435-2HG	0.3722	2.0145	2.4362	9.95E-23	8.04E-22
HCG18	0.3418	0.9249	1.4363	2.01E-18	6.95E-18
PAXIP1-AS2	0.8206	1.1892	0.5352	5.38E-04	6.14E-04
LINC01857	0.2596	0.5341	1.0408	2.05E-04	2.46E-04
AC055822.1	0.2216	0.7043	1.6681	4.86E-14	9.25E-14
USP46-AS1	0.6233	1.1040	0.8248	7.88E-09	1.06E-08
SNHG20	0.3618	1.2887	1.8327	3.89E-24	4.83E-23
LENG8-AS1	0.1903	0.8826	2.2133	1.87E-24	3.35E-23
LINC02035	0.2913	0.6189	1.0872	1.71E-10	2.55E-10
SNHG17	1.4286	3.7033	1.3742	3.19E-16	8.15E-16
AL358472.2	0.3899	0.8929	1.1956	4.37E-16	1.03E-15

AC115618.2	2.2914	4.7024	1.0372	5.94E-11	9.61E-11
ZNF529-AS1	0.2941	0.9142	1.6362	1.19E-20	5.51E-20
SNHG32	9.7545	36.2081	1.8922	3.75E-25	9.09E-24
LINC00847	2.0986	4.2641	1.0228	3.39E-16	8.44E-16
AC110285.2	0.2284	1.5395	2.7530	3.56E-19	1.44E-18
AC002398.1	0.3100	0.8941	1.5284	4.96E-15	1.02E-14
AC097359.2	0.5025	1.0275	1.0321	4.35E-13	7.96E-13
HCP5	3.0803	8.0969	1.3943	1.13E-09	1.59E-09
LINC01278	1.1904	2.2709	0.9318	3.53E-16	8.57E-16
AC008764.2	0.4210	1.1844	1.4924	8.15E-11	1.26E-10
GIHCG	0.3891	1.9250	2.3066	3.99E-24	4.83E-23
NNT-AS1	1.0401	1.8696	0.8460	3.22E-12	5.48E-12
SMIM25	0.8884	0.6370	(0.4800)	3.20E-09	4.37E-09
AC009779.3	1.7933	3.7839	1.0772	1.42E-16	3.73E-16
AC024075.1	0.1747	0.5499	1.6546	5.20E-10	7.65E-10
SNHG21	0.1935	0.6422	1.7303	6.92E-21	3.53E-20
CTBP1-DT	0.7380	1.7976	1.2844	1.00E-21	6.65E-21
PTOV1-AS1	0.4732	1.2320	1.3805	4.84E-23	4.27E-22
MID1IP1-AS1	0.3221	1.0101	1.6490	2.55E-09	3.54E-09
NRAV	0.8204	2.0907	1.3496	3.98E-19	1.54E-18
LINC00342	0.3893	1.1316	1.5395	2.17E-14	4.30E-14
AC067852.2	1.0183	1.7592	0.7888	8.56E-17	2.37E-16
AGAP2-AS1	0.7056	1.3548	0.9413	2.73E-01	2.82E-01
AC046143.2	0.4691	0.8824	0.9116	2.77E-04	3.27E-04
AC060766.4	0.3777	0.6681	0.8229	4.76E-04	5.49E-04
C2CD4D-AS1	1.0284	4.6383	2.1732	5.25E-19	1.96E-18
WAC-AS1	2.9567	5.8393	0.9818	7.17E-16	1.65E-15
LINC01836	0.2292	0.8788	1.9389	4.29E-03	4.67E-03
FAM111A-DT	0.2731	0.6983	1.3543	7.48E-17	2.20E-16

AC068987.3	0.2685	1.6051	2.5798	5.60E-14	1.04E-13
RAD51-AS1	0.3433	1.0565	1.6219	7.47E-20	3.15E-19
AC007406.5	0.7257	1.1290	0.6376	7.14E-06	8.66E-06
LINC00909	0.7944	1.4567	0.8747	1.20E-12	2.12E-12
PCED1B-AS1	0.5766	1.0272	0.8332	1.09E-02	1.16E-02
AC010547.2	2.3158	1.1320	(1.0327)	5.12E-24	5.52E-23
NORAD	23.3232	35.8956	0.6220	2.03E-11	3.34E-11
LINC01436	0.0886	1.6908	4.2540	5.70E-06	7.00E-06
AP000759.1	0.6051	1.5374	1.3452	3.18E-14	6.17E-14
AL606489.1	0.0559	0.8343	3.8987	8.23E-24	7.99E-23
SNHG14	0.2114	0.6378	1.5934	6.13E-03	6.61E-03
AL450326.1	0.9618	1.0678	0.1509	8.48E-01	8.48E-01
AC067838.1	0.5154	0.8691	0.7540	3.72E-04	4.35E-04
AC125257.1	1.2604	2.6888	1.0931	1.38E-21	8.38E-21
AL162595.1	0.2495	0.7704	1.6268	1.36E-17	4.31E-17
AL391121.1	0.6260	1.0145	0.6966	7.19E-04	8.02E-04
MAPKAPK5-AS1	1.3507	3.9676	1.5545	8.83E-27	5.16E-25
ANKRD10-IT1	0.8534	2.2690	1.4108	1.66E-07	2.18E-07
AL035461.3	0.3701	2.0595	2.4762	6.52E-20	2.87E-19
CASC19	0.6461	1.0941	0.7598	3.60E-01	3.68E-01
OIP5-AS1	5.0180	6.4319	0.3581	9.14E-04	1.01E-03
CYTOR	0.7227	4.1307	2.5148	8.43E-21	4.09E-20
EBLN3P	3.9468	5.9949	0.6031	3.52E-06	4.37E-06
AC000123.1	0.4334	0.9597	1.1469	1.65E-12	2.86E-12
AP001318.2	1.6202	2.3612	0.5434	2.92E-07	3.78E-07
AC009237.14	1.3384	3.0172	1.1727	1.64E-15	3.54E-15
THAP9-AS1	1.3603	2.5277	0.8939	7.67E-11	1.20E-10
AP003486.1	0.3416	0.8274	1.2765	1.61E-15	3.54E-15
AC104083.1	1.8080	1.7737	(0.0276)	5.46E-04	6.16E-04

SNHG16	1.8771	2.9914	0.6723	2.89E-06	3.64E-06
AC005288.1	2.9480	5.2976	0.8456	7.18E-11	1.14E-10
AC073611.1	0.2047	0.7330	1.8400	1.15E-18	4.13E-18
AC074117.1	0.4727	1.2945	1.4535	4.76E-21	2.57E-20
NRSN2-AS1	0.3614	0.9529	1.3986	4.25E-12	7.11E-12
FGD5-AS1	10.2824	16.3496	0.6691	1.13E-09	1.59E-09
EIF3J-DT	0.4691	1.0156	1.1143	7.32E-16	1.65E-15
AL035071.1	0.8036	1.9988	1.3146	6.45E-15	1.30E-14
AC005586.1	0.2915	0.8671	1.5726	8.31E-13	1.49E-12
AC011451.1	0.5891	0.8046	0.4497	2.55E-02	2.69E-02
AC005332.5	0.3598	1.7910	2.3156	1.06E-26	5.16E-25
AL354836.1	0.4877	1.4962	1.6173	2.75E-15	5.80E-15

Suppl Table 4 Vascular invasion-related lncRNAs correlated with prognosis

LncRNAs	conMean	treatMean	logFC	*P*-Value	FDR
LINC00205	0.2893	1.4056	2.2803	1.03E-21	6.65E-21
AC099850.4	0.1832	2.0816	3.5064	3.81E-18	1.27E-17
AC048341.2	0.2150	1.0879	2.3390	3.03E-21	1.73E-20
MIR4435-2HG	0.3722	2.0145	2.4362	9.95E-23	8.04E-22
LENG8-AS1	0.1903	0.8826	2.2133	1.87E-24	3.35E-23
AC110285.2	0.2284	1.5395	2.7530	3.56E-19	1.44E-18
GIHCG	0.3891	1.9250	2.3066	3.99E-24	4.83E-23
C2CD4D-AS1	1.0284	4.6383	2.1732	5.25E-19	1.96E-18
AC068987.3	0.2685	1.6051	2.5798	5.60E-14	1.04E-13
LINC01436	0.0886	1.6908	4.2540	5.70E-06	7.00E-06
AL606489.1	0.0559	0.8343	3.8987	8.23E-24	7.99E-23
AL035461.3	0.3701	2.0595	2.4762	6.52E-20	2.87E-19
CYTOR	0.7227	4.1307	2.5148	8.43E-21	4.09E-20
AC005332.5	0.3598	1.7910	2.3156	1.06E-26	5.16E-25

Suppl Table 5 Vascular invasion -related lncRNA pairs with prognostic value

LncRNA pairs	HR	HR.95L	HR.95H	pvalue
LINC00205|AC099850.4	0.5943	0.4160	0.8489	0.0042
AC099850.4|AC048341.2	1.5128	1.0470	2.1859	0.0275
AC099850.4|MIR4435-2HG	1.4300	1.0135	2.0177	0.0417
AC099850.4|LENG8-AS1	1.4841	1.0192	2.1610	0.0395
AC099850.4|AC110285.2	1.5608	1.0929	2.2290	0.0144
AC099850.4|AC005332.5	1.4796	1.0467	2.0917	0.0265
AC048341.2|LENG8-AS1	0.6620	0.4658	0.9409	0.0215
AC048341.2|GIHCG	0.5866	0.3744	0.9193	0.0200
AC048341.2|AC068987.3	0.6897	0.4819	0.9869	0.0422
AC048341.2|LINC01436	0.5836	0.4011	0.8491	0.0049
MIR4435-2HG|AC110285.2	1.5576	1.0568	2.2957	0.0251
AC110285.2|GIHCG	0.6748	0.4578	0.9946	0.0469

Suppl Table 6 Vascular invasion -related lncRNA pairs signature

DELncRNAs pairs	coef	HR	HR.95L	HR.95H	*P*value
AC099850.4|MIR4435-2HG	0.3776	1.4587	1.0188	2.0886	0.0392
AC048341.2|LENG8-AS1	-0.3176	0.7279	0.5051	1.0489	0.0884
AC048341.2|GIHCG	-0.3402	0.7117	0.4468	1.1335	0.1521
AC048341.2|LINC01436	-0.3572	0.6996	0.4748	1.0308	0.0708
MIR4435-2HG|AC110285.2	0.4547	1.5756	1.0440	2.3779	0.0304
